# The impact of HPV vaccine narratives on social media: Testing narrative engagement theory with a diverse sample of young adults

**DOI:** 10.1016/j.pmedr.2022.101920

**Published:** 2022-07-22

**Authors:** Amy E. Leader, Michelle Miller-Day, Rikishi T. Rey, Preethi Selvan, Anne E. Pezalla, Michael L. Hecht

**Affiliations:** a Division of Population Science, Department of Medical Oncology, Thomas Jefferson University, United States; b School of Communication, Chapman University, United States; c Department of Communication, Clemson University, United States; d REAL Prevention, LLC, United States; e Department of Psychology, Macalester College, United States

**Keywords:** Human papillomavirus (HPV) vaccination, Narrative engagement theory, Health communication, Social media

## Abstract

Rates of human papillomavirus (HPV) infection are highest in young adults, who can be vaccinated against HPV if they were not vaccinated as adolescents. Since young adults increasingly access health information on social media, we tested the impact of a social media campaign with narrative-based health information on intentions related to HPV vaccination. We also aimed to understand which ads resonated most with young adults and led to higher survey completion rates. We created social media posts featuring videos promoting HPV vaccination. We launched a sponsored ad campaign on Facebook to reach young women, ages 18–26, across the country. Participants were randomly assigned one of 6 videos and then completed a brief survey about video engagement and intentions to: talk with a health care professional, talk with friends or family, and vaccinate against HPV. A descriptive correlational design and a test for moderation were used to explore hypothesized relationships. Across all ads, 1332 link clicks led to 991 completed surveys that were reduced to 607 surveys (95 % ages 18–26, 63 % non-Caucasian; 58 % sexually active). Higher video engagement was associated with stronger intentions to talk with a health care professional (*r* = 0.44, *p* =.01), talk with friends/family (*r* = 0.52, *p* =.01), and vaccinate against HPV (*r* = 0.43, *p* =.01). Young adults were receptive to watching narrative-based health information videos on social media. When promoting HPV vaccination, more engaging information leads to greater intentions to talk about the vaccine and get vaccinated.

## 1 Background

### 1.1 Human papillomavirus and the vaccine

The human papillomavirus (HPV) is the most common sexually transmitted infection in the United States. (CDC, 2021) One in four U.S. adults will be infected with HPV during their lifetime. (Chesson et al., 2014) HPV incidence rises with increasing age and is highest among individuals in their late teens and early twenties. (Viens et al., 2016) Risk factors for HPV infection are associated with having a higher number of lifetime sexual partners and initiating sex at a young age. (Osazuwa-Peters et al., 2019) HPV causes virtually all cervical and anal cancers, a majority of oropharyngeal and vaginal cancers, half of vulvar cancers, and one-third of penile cancers. (Lowy and Schiller, 2012) HPV also causes genital warts. (Park et al., 2015).

The HPV vaccine is recommended for adolescents between the ages of 11 and 12 years, when immunogenicity is highest and previous infection with HPV is rare. (Meites et al., 2019) While the vaccine is approved for those up to age 45, (Food and Drug Administration, 2018) the Advisory Committee on Immunization Practices (ACIP) recommends vaccination through age 26. (Meites et al., 2019) Despite the safety and efficacy of the HPV vaccine (Arbyn and Xu, 2018, St Laurent et al., 2018), uptake has been suboptimal compared to other routine vaccinations. (Elam-Evans et al., 2020) For young adults aged 18–26 years, the HPV vaccine initiation rate is 39 % and the completion rate is 22 %. (Boersma and Black, 2020).

There is a small but growing body of evidence about HPV vaccine acceptability and uptake in young adults ages 18 through 26. The earliest studies focused on college students. (Daley et al., 2010, Patel et al., 2012, Marchand et al., 2012) A more recent population-based study of 18- to 26-year-olds found that men, those with a high school diploma or less education, and those born outside the United States were less likely to initiate and complete the HPV vaccine series. (Adjei Boakye et al., 2018).

Understanding effective messages is crucial to vaccine uptake. Numerous national organizations have campaigns to promote HPV vaccination, typically focusing on parents. (American Cancer Society. Mission: HPV Cancer Free. Available at: https://www.cancer.org/healthy/hpv-vaccine.html. Last accessed on February 19, 2019, Accelerating, 2014, The National, 2019, Academy, 2019) Messages that appeal to young adults who are not parents will need to be different than those created for parents. First, messages will need to focus on vaccinating oneself rather than one’s child. Messages may stress the sexually transmitted nature of the virus, which is salient for adults but has been downplayed for parents. Vaccination messages for young adults may have the look and feel of adult STI prevention campaigns, which are often more blunt, edgier, and appealing to an adult audience. (Parenthood and Tools, 2021).

### 1.2 Theoretical Framework: A Narrative-Based approach to health promotion

Narrative engagement theory (Miller-Day and Hecht, 2013) provides a useful framework for message design to promote HPV vaccination. (Hecht et al., 2021, Hopfer et al., 2018, Hopfer and Clippard, 2011) Miller-Day and Hecht define narrative as talk organized around significant experiences, with characters undertaking action, within a context, with implicit or explicit beginning and end points and significance for the narrator or her or his audience. (Miller-Day and Hecht, 2013) Personal narratives are culturally grounded and can provide valuable insight into understanding health decisions. (Kreuter et al., 2007) Translating those insights into prevention messages can be engaging and effective, create more appealing messaging, and extend reach to low awareness and/or resistant audiences (Miller-Day and Hecht, 2013, Kreuter et al., 2007, Larkey and Hecht, 2010). Vaccine narratives translated into an intervention can be particularly useful with people who have difficulty understanding statistics used in health messages; (Petraglia, 2007) provide modeling of behaviors enhancing self-efficacy; (Miller-Day and Hecht, 2013) highlight the potential benefits of a health behavior, targeting beliefs and norms about health behaviors; (Kreuter et al., 2007) and be a vehicle to “re-story” or change an existing HPV vaccination narrative to promote health behavior change. (Hecht et al., 2021, De Oliveira et al., 2019, Gerend and Shepherd, 2012) Narrative engagement theory predicts that narratives can provide the content for vaccine messaging that is more likely to be relevant to and resonate with young people (Hecht and Krieger, 2006, Hecht et al., 2009) and effectively engage an audience when considering health behavior. (Miller-Day and Hecht, 2013, Larkey and Hecht, 2010, Gonzalez Cabreram and Igartua, 2018).

### 1.3 Study purpose

The purpose of this social media-based intervention study was to test whether variations in narrative engagement led to differences in HPV vaccine intentions. While there is a growing evidence base of the impact of interventions on social media, (Asare et al., 2021) this is one of the first to be grounded in narrative engagement theory. We were interested in recruiting as diverse of an audience as possible; hence, we used this opportunity to simultaneously test the most effective messaging for recruiting through social media. We proposed the following research questions:

RQ1: Do HPV videos with higher levels of narrative engagement have a stronger effect on intentions to talk about the HPV vaccine and be vaccinated against HPV than less engaging HPV videos?
RQ2: Do perceptions of the COVID pandemic moderate the effects of video engagement on vaccine intention?
RQ3: Which social media ad campaign, and corresponding ad features, was most likely to lead to survey completion?

## 2 Methods

### 2.1 Overview

Our goal was to engage an audience of young adult women to watch narrative-based videos addressing HPV vaccination and complete a survey about their engagement with the videos, their intentions to talk to others about HPV vaccination, and their own HPV vaccination intentions. We recruited via social media, where potential participants saw a sponsored ad that we created for the study. Contained within the ad was a link; clicking the link took participants to a randomly assigned, narrative-based video on HPV vaccination. After watching the video, participants completed a short survey. Participants were compensated $10 via an electronic gift card for completing the survey. This study was deemed exempt by the organization’s Institutional Review Board.

### 2.2 Narrative-Based video intervention development

We used six narrative-based videos designed to increase HPV vaccination rates among adult women (mean video length = 1 min, 24 s). These videos focused on vaccinating oneself rather than one’s child and stressed the sexually transmitted nature of the virus and its link to various cancers. The videos were evidence-based and part of the previously developed “HPV Wellness Suite: Women’s Stories” HPV intervention. (Hecht et al., 2021, Hopfer et al., 2018, Hopfer et al., 2017) The videos have proven efficacious and are described elsewhere in two clinic-based randomized clinical trials. (Hecht et al., 2021, Hopfer, 2012) In general, this approach involves in-depth interviews to solicit vaccine decision narratives, the details and events of these decisions serve as the foundation of the prototypical stories depicted in the videos. (Larkey and Hecht, 2010) Health facts, positive modeling of the behavior, and a call to action are then woven into the story. Descriptions of the videos can be found in the Appendix. Copies of the video are available upon request.

### 2.3 Social media advertisement development

To recruit participants on social media to complete the survey, we created 15-second video ads that depicted mostly young women but could appeal to all women, given that the vaccine is approved for women up to age 45. The first video depicted general images of women in a slideshow format. The second video depicted 18–26-year-old male and female adults interacting with each other. The two video ads were paired with two different messages: a statement, “The HPV Vaccine Has Been Proven to Prevent Cancer in Women,” or a question, “Did you know the HPV Vaccine Prevents Cancer in Women?” We also created four static (single image) ads. The first two ads featured an image of a young Asian adult female, while the second two ads featured an image of a young African American adult female. The static ads were paired with the same messages that were used in the video ads. Facebook reviews all ads prior to distributing the ads across their platform. The third static add was rejected because it was labeled a social issue. The team decided not to pursue an advanced review and therefore it was not published. The full set of ads are in Table 1.

**Table 1:** Campaign Creatives.

### 2.4 Recruitment

The study ran from August 12, 2020 through September 26, 2020 on Instagram platforms (Feed, Explore, and Stories) using Facebook (FB) Ad Manager. We chose Instagram platforms because 18–26-year-olds are more likely to use Instagram rather than Facebook. (Pew Research Center, 2021) We used the recruitment period as a time to test the effectiveness of the ads on their ability to lead to completed surveys. Those who had previously been vaccinated against HPV, determined through self-report, were excluded from participating in the study. Three different campaigns (ad + message) were conducted (Table 2). In the first campaign, we tested the message framing (statement vs question) and kept the video constant. The ads were tested against each other using the A/B Testing feature within FB Ad Manager. The second campaign focused on testing two different images in combination with either type of ad message (Table 1: Video Ads A & B vs C & D). For the third campaign, we switched to the static ads and added primary text, (“Learn more. Take a survey. Get an Amazon gift card on us.”). This campaign was continued through the end of the study without any further changes.

**Table 2:** Facebook Analytic Data for Each Campaign.

**Campaign**	**I** (Message Testing)		**II** (Image Testing)				**III** (Message and Image Testing)		
**Dates**	8/12 – 8/23		8/25 – 8/29		8/25 – 8/29		9/10 – 9/26		
**Creatives Used**	**A**	**B**	**A**	**B**	**C**	**D**	**Static Ad 1**	**Static Ad 2**	**Static Ad 4**
**Reach**	**A:** 8,741	**B:** 7,527	**A:** 8,732	**B:** 4,964	**C:** 5,006	**D:** 5,244	**1:** 21,416	**2:** 6,666	**4:** 15,317
**Impression**	**A:** 10,115	**B:** 8,841	**A:** 10,535	**B:** 5,733	**C:** 5,851	**D:** 6,123	**1:** 29,355	**2:** 7,741	**4:** 19,243
**Video Percentage Watched:**	**A:** 6.7%	**B:** 6.1%	**A:** 5.25%	**B:** 5.74%	**C:** 6.65%	**D:** 6.84%	--	--	--
**Link Clicks (Unique Link Clicks):**	**A:** 18 (18)	**B:** 25 (25)	**A:** 13 (13)	**B:** 20 (20)	**C:** 12 (11)	**D:** 15 (15)	**1:** 515 (412)	**2:** 248 (215)	**4:** 771 (623)
**Number of Completed Surveys**	3		1				987		
**Cost per Result**	**A:** $1.33	**B:** $0.96	**A:** $1.86	**B:** $0.78	**C:** $1.67	**D:** $1.32	**1:** $0.61	**2:** $0.57	**4:** $0.59

*Ad 3 was rejected by Facebook.

### 2.5 Survey measures

#### 2.5.1 Engagement

The engagement variable measurement assessed interest, realism, and identification. (Lee et al., 2011) Items were measured on Likert-type scale (1 = *strongly disagree*; 5 = *strongly agree*). Interest items included, “The video was interesting,” “I paid attention to the video,” “when watching the video, I did NOT think about other things,” “I had a hard time keeping my mind on the video” (reverse coded), “If this video was available on Instagram, I would share it,” and “The video was boring” (reverse coded). Realism items consisted of, “The video seemed realistic to me” and “the video was believable.” Identification items were, “The people in the video seemed like people I know” and “The people in the video seemed like me.” This scale obtained a reliable Cronbach alpha (α = 0.85, *M* = 3.78, *SD* = 0.60).

#### 2.5.2 Dependent variables

Dependent variables identified participants’ intentions to talk with a healthcare provider or their friends or family about the vaccine or to receive the HPV vaccine the next time they visited their doctor. Each item was measured as a single item on a 5-point Likert-type scale (1 = *very unlikely*, 5 = *likely*). Items included, “I intend to talk with my healthcare professional about the HPV vaccine” (*M* = 3.86; *SD* = 1.04), “I intend to talk to my friends/family about the HPV vaccine” (*M* = 3.62; 1.14; *SD* = 1.14), and “I intend to get the HPV vaccine the next time I see my doctor” (*M* = 3.68; *SD* = 1.10).

#### 2.5.3 Moderating variable

To address Research Question 2, to understand if perceptions of the COVID-19 pandemic moderated the effects of video engagement on vaccine intention, the covariate regarding COVID-19 “COVID has impacted my decision to get the HPV vaccine” (*M* = 2.91, *SD* = 1.27) was measured on a Likert-type scale (1 = *strongly disagree,* 5 = *strongly agree*).

### 2.6 Data management and statistical analysis

Surveys were deployed through Qualtrics, where data was collected and stored as a secure datafile. To test differences in engagement and intentions by video, we used a two-tailed Pearson correlation and an analysis of variance (ANOVA). Research Question 2, that perceptions of the COVID-19 pandemic (“COVID has impacted my decision to get the HPV vaccine”) would moderate the effect of video engagement on vaccine intentions, was answered using Model 1 from the PROCESS macro in SPSS. (Hayes, 2018) Models used percentile bootstrapped standard errors and 95 % confidence intervals from 5,000 resamples to examine the indirect effects. Continuous variables were standardized before being entered into the model, making coefficients partially standardized. Confidence intervals not containing zero were interpreted as statistically significant.

### 2.7 Social media campaign analytics

Campaign ad performance was measured by using a number of metrics that were available through Facebook’s Ad Manager. ‘Reach’ was used to track the number of Instagram users who saw the ads at least once. ‘Impressions’ measured the number of times our ads were on screen, which included multiple views by the same user. ‘Link clicks’ and ‘unique link clicks’ were used as the main metric for measuring performance and included any Instagram user that clicked on the link in our ads. Cost per result indicated the cost per link click based on the total amount spent on the campaign. Research Question 3, which ad and ad features led to the greatest number of completed surveys, was determined by a frequency count of the number of surveys completed stemming from each ad.

## 3 Results

### 3.1 Social media campaign (RQ3)

The results of the four campaigns can be seen in Table 2. In the first campaign, we found no differences in the number of link clicks between the two video ads. We found that 43 link clicks translated into 3 completed surveys (Table 2). A/B testing showed no difference between the type of message (statement vs question) used in the campaign. The second campaign with video ads resulted in 1 additional completed survey from 59 link clicks. After the change to static ads, the third and final campaign resulted in 987 surveys from 1,250 link clicks and averaged about $0.59 per link click ($0.92 per completed survey).

### 3.2 Participants

**Fig. 1:**
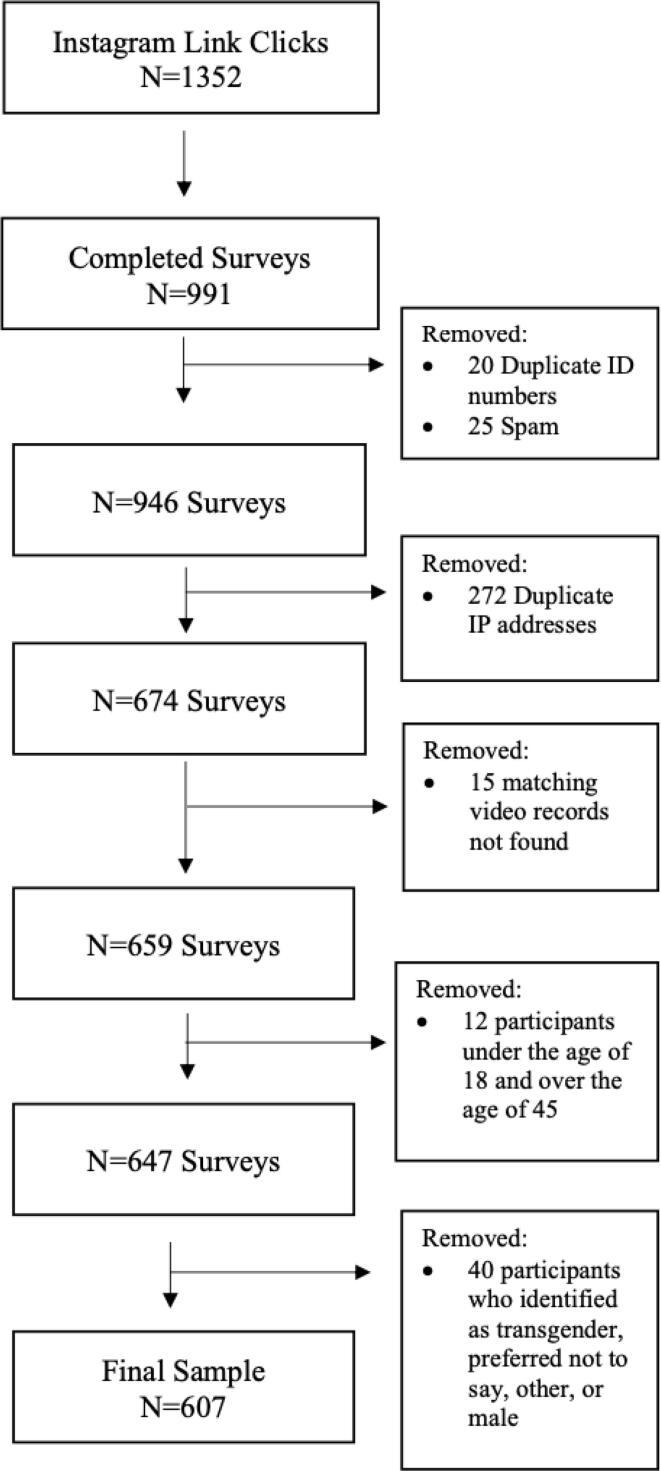
CONSORT Diagram.

Across all ad campaigns, 1332 link clicks led to 991 completed surveys and 607 usable surveys (Fig. 1). A full description of the 607 participants is in Table 3. Most participants (*n* = 579, 95.4 %) were between the ages of 18–26, 23 (3.8 %) were 27–34, and 5 (0.8%) were between the ages of 35–45. The sample was diverse with 226 (37.2 %) who identified as white/Caucasian, 160 (26.4 %) Asian/Asian American, 88 (14.5 %) Hispanic/Latino/Latina, and 59 (9.7 %) Black/African American. The number of participants who were randomized to each narrative engagement video ranged from 93 (Video 5) to 107 (Video 2).

**Table 3:** Participant Demographics.

**Demographic**	**N**	**%**
*Gender*		
Female	607	100
*Age*		
18–26	579	95.4
27–34	23	3.8
35–45	5	0.8
*Ethnicity*		
White/Caucasian	226	37.2
Asian/Asian American	160	26.4
Hispanic/Latino/Latina	88	14.5
Black/African American	59	9.7
More than one ethnicity	39	6.4
Other	35	5.8
*Sexual Activity*		
Sexually active in the past 3 months	353	58.2
Not sexually active in the past 3 months	254	41.8

### 3.3 Dependent variables (RQ1)

Engagement scores for each of the six videos ranged from the lowest at 3.62 (SD = 0.65) for Video 2 to the highest at 3.89 (SD = 0.60) for Video 1 (Table 4). Engagement scores were significantly higher for Video 1 and Video 5 compared to Video 2. (Table 5).

**Table 4:** Mean Scores of Each Video on Key Outcome Measures.

			**Video**											
	Composite Score		1		2		3		4		5		6	
*N*			100		107		102		111		93		94	
	*M*	*SD*	*M*	*SD*	*M*	*SD*	*M*	*SD*	*M*	*SD*	*M*	*SD*	*M*	*SD*
Engagement* (α = 0.85)	3.78	0.60	3.89	0.6	3.62	0.65	3.78	0.58	3.82	0.59	3.88	0.51	3.81	0.65
I intend to talk to healthcare professional about the HPV vaccine	3.54	0.92	3.88	1.09	3.85	0.99	4.03	0.96	3.86	0.89	3.83	1.07	3.9	1.13
I intend to talk to friends/family about the HPV vaccine	3.65	1.12	3.55	1.25	3.63	1.12	3.85	0.97	3.67	0.97	3.56	1.17	3.61	1.25
I intend to get the HPV vaccine the next time I see my doctor	3.71	1.08	3.63	1.19	3.75	1.00	3.84	1.03	3.63	1.04	3.62	1.06	3.76	1.18
Covid-19 has impacted my decision to get the HPV vaccine	2.91	1.27	2.85	1.27	3.09	1.21	3.34	1.35	2.76	1.23	2.82	1.28	2.87	1.25

*Note:* Video 1: Young Woman at Clinic; Video 2: African American Provider-Patient Caucasian woman; Video 3: Women's Stories Truck; Video 4: Mom Daughter Kitchen; Video 5: Young Adult Overview; Video 6: Benefits of vaccination.

*Engagement is a summary score of all the engagement items.

**Table 5:** Differences in Engagement and Intentions by Video.

	*n*	Engagement* (*M, SD)*	I intend to talk with my healthcare professional about the HPV vaccine *(M, SD)*	I intend to talk to my friends/family about the HPV vaccine *(M, SD)*	I intend to get the HPV vaccine the next time I see my doctor *(M, SD)*	I intend to share the Instagram post *(M, SD)*
*F*		2.91	0.51	0.98	0.73	0.25
*p*		0.01	0.77	0.43	0.6	0.94
η^2^		0.02	0.004	0.008	0.006	0.004
Video 1: Provider-Patient Story at Clinic #1	107	3.89, 0.60	3.88, 1.9	3.63, 1.12	3.75, 1.00	2.96, 1.34
Video 2: Mother-Daughter Story	111	3.62, 0.65	3.86, 0.89	3.67, 0.97	3.63, 1.04	2.86, 1.22
Video 3: Rural Location Story	102	3.78, 0.58	4.03, 0.96	3.85, 0.97	3.84, 1.03	3.02, 1.23
Video 4: Story Montage #1	94	3.82, 0.59	3.90, 1.13	3.62, 1.25	3.76, 1.18	2.86, 1.24
Video 5: Montage #2	93	3.88, 0.51	3.83, 1.97	3.56, 1.17	3.62, 1.06	2.97, 1.33
Video 6: Provider-Patient Story at Clinic #2	100	3.81, 0.65	3.88, 1.09	3.55, 1.25	3.63, 1.19	2.91, 1.25

* Summary Score of Engagement Items.

Across all videos, the mean score was highest for “I intend to talk with my healthcare professional about the HPV vaccine” (*M* = 3.89, *SD* = 1.02) and lowest for “I intend to talk to my friends/family about the HPV vaccine” (*M =* 3.65, *SD* = 1.12). The mean score for “I intend to get the HPV vaccine the next time I see my doctor” was 3.71 (*SD* = 1.08). (Table 4).

Results of a two-tailed Pearson correlation to test whether videos with a higher narrative engagement had stronger effects on intentions indicated that engagement was positively associated with intentions to talk with a healthcare professional about the HPV vaccine (*r* = 0.44, *p* =.01) and intentions to talk to friends/family about the HPV vaccine (*r* = 0.52, *p* =.01). Results of a two-tailed Pearson correlation indicated that there was also a positive relationship between engagement and intentions to vaccinate (*r* = 0.43, *p* =.01). Therefore, the hypothesis was supported (Table 6). When examining if there was a significant difference in any of the intention variables between the different videos, the ANOVA was not significant for any of them: “I intend to talk with my healthcare professional about the HPV vaccine” *F*(5, 601) = 0.51, *p* =.77, (η^2^ = 0.004), “I intend to talk to my friends/family about the HPV vaccine” *F*(5, 601) = 0.98, *p* =.43, (η^2^ = 0.008), and “I intend to get the HPV vaccine the next time I see my doctor” *F*(5, 601) = 0.73, *p* =.60, (η^2^ = 0.00) (Table 5).

**Table 6:** Correlation Matrix for Engagement and Intention Items.

	1	2	3	4
1. Engagement*	–			
2. I intend to talk with my healthcare professional about the HPV vaccine	0.442^**^	–		
3. I intend to talk to my friends/family about the HPV vaccine	0.516^**^	0.626^**^	–	
4. I intend to get the HPV vaccine the next time I see my doctor	0.431^**^	0.757^**^	0.554^**^	–

*Summary score of all engagement items.

**Correlation is significant at the 0.01 level (2-tailed).

### 3.4 Moderation (RQ2)

**Fig. 2:**
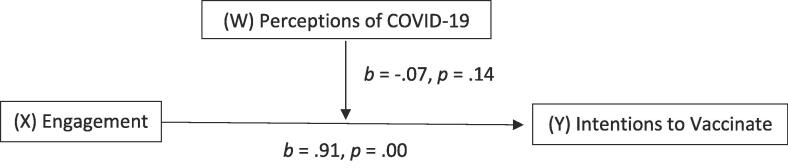
Model 1 Conceptual Diagram.

Results of a moderation analysis indicated that “COVID has impacted my decision to get the HPV vaccine” (M = 2.91, SD = 1.27) (Table 4) and the overall model (i.e., perceptions of the COVID-19 pandemic moderating the relationship between engagement and intentions) was significant, *F* (3, 603) = 60.32, *p* <.001, *R*^2^ = 0.23. However, the interaction effect between engagement and the impact that COVID-19 has on vaccine intentions was not significant *B* = -0.07, *SE =* 0.05, *t* = -1.49, *p* =.14, 95 % CI [-0.17, 0.02]. (Fig. 2). Thus, COVID appears to have an independent but not moderating effect on the engagement to intentions pathway.

## 4 Discussion

The purpose of this study was threefold: to determine if videos with higher levels of engagement had higher levels of intention to talk with someone about the vaccine or to be vaccinated, to determine if certain videos resonated more strongly with young adults recruited through social media, and to determine if perceptions about the COVID-19 pandemic would impact intentions to vaccinate against HPV.

First, we found that overall videos that scored higher on engagement evoked stronger intentions to vaccinate. This is consistent with, but an extension of, previous research conducted with the WS video intervention. For example, Rey et al. reported comparable levels of engagement and a strong association between engagement and video persuasiveness, but did not evaluate vaccination intentions. (Rey et al., 2021) Hecht et al. measured the effects of a clinic based WS video intervention on vaccine intentions and vaccine self-efficacy and found significant positive intervention effects on each, but did not examine engagement with the intervention videos. (Hecht et al., 2021) Promisingly, when looking at the effect of all videos across participants, there was an association between video exposure and vaccine intentions. This confirms predictions from Narrative Engagement Theory about the types of videos that are effective in promoting behavior change.

The findings from this study also have implications for promoting research studies on social media. First, we found that static ads were more effective than video clips in attracting our audience to the study. This may be the nature of the social media environment, where people are rapidly viewing large quantities of content and do not intend to view any one post for a substantial amount of time. Being able to move quickly to the study rather than watching a short video was preferred for our audience of young adults. We also confirmed that recruiting through social media led to diverse representation of participants. In our sample, about two-thirds of participants identified with a racial or ethnic minority group. This is reflective of other studies that have had success in recruiting diverse participant samples through social media. (Whitaker et al., 2017).

Our study also contributes to knowledge about perceptions of vaccination during COVID-19. Access to preventive care, like the HPV vaccine, was halted in many communities, resulting in steep declines in routine vaccination. (Findling et al., 2020, Patel Murthy et al., 2021) We wondered if perceptions of the COVID pandemic moderated the effects of video engagement on vaccine intention. The findings of this study suggest that perceptions about COVID-19 did not moderate the effects of the intervention on intentions to vaccinate. This illustrates that the COVID-19 pandemic has not impacted the HPV vaccination decision-making process. Even as the COVID-19 pandemic continues, primary care physicians, as well as other health care professionals such as pharmacists, should continue to have conversations with their patients about the importance of being vaccinated against HPV.

It is important to still be vigilant when promoting HPV vaccination. Currently, social media is a breeding ground for misinformation and disinformation, particularly as it relates to health information. (Wang et al., 2019) The public health and medical communities have a responsibility to counterbalance this information in a way that is engaging and credible to audiences. It is not enough to assume that the visibility on social media is enough to inspire change in its viewers. Indeed, considering how anyone posting on social media must jockey for space with an almost-limitless number of other posts, which may contain misinformation, any credible, pro-vaccine message must stand out. Ads like the ones we created, as well as videos such as the *HPV Wellness Suite: Women’s Stories,* can reverse this trend by providing high-quality, factually accurate information on social media. Moreover, the results of the study can inform future public health initiatives to increase HPV vaccination among an older age group, those over the age of 18, who are making vaccination decisions for themselves rather than relying on a parent.

While there were numerous strengths to this study, such as using a theoretically grounded, evidence-based video intervention, there were limitations. Due to the nature of the study, we were unable to measure actual HPV vaccination behavior. While self-report of HPV vaccination status has been shown to be an acceptable measure of actual vaccination behavior, (Yamaguchi et al., 2018, Rolnick et al., 2013) and was most feasible in this study, it is not error-free. There may also be a social desirability bias to over-report intentions or positive attitudes toward vaccination. We only posted on Facebook-based platforms and are unable to generalize these findings to other social media platforms. It should be noted that Facebook Ad Manager does not provide individual level analytic data. The study was designed to appeal to young women; it is unclear whether a similar type of study and intervention would appeal to young men, who are also eligible for HPV vaccination. Last, while we saw significant differences in engagement between videos, the differences were small and may not be conceptually meaningful. The variation in responses was also small and may be indicative of a homogeneous sample of participants who already had positive views of the HPV vaccine. Nevertheless, this study lays the foundation for future research and interventions that aim to promote HPV vaccination on social media, in understanding how to reach key and diverse audiences with messages that are both credible and engaging.

## Funding statement

Funding was supported by the Grant or Cooperative Agreement Number, 5 R44DP006291-03, by the Centers for Disease Control and Prevention. Its contents are solely the responsibility of the authors and do not necessarily represent the official views of the Centers for Disease Control and Prevention or the Department of Health and Human Services.

## CRediT authorship contribution statement

**Amy E. Leader:** Conceptualization, Methodology, Visualization, Writing – original draft, Writing – review & editing, Project administration. **Michelle Miller-Day:** Conceptualization, Methodology, Writing – original draft, Writing – review & editing. **Rikishi T. Rey:** Conceptualization, Methodology, Formal analysis, Data curation, Visualization, Writing – original draft, Writing – review & editing. **Preethi Selvan:** Conceptualization, Methodology, Software, Formal analysis, Data curation, Visualization, Writing – original draft, Writing – review & editing. **Anne E. Pezalla:** Conceptualization, Methodology, Writing – original draft, Writing – review & editing. **Michael L. Hecht:** Conceptualization, Methodology, Resources, Writing – original draft, Writing – review & editing, Funding acquisition.

## Declaration of Competing Interest

The authors declare that they have no known competing financial interests or personal relationships that could have appeared to influence the work reported in this paper.

## Appendix

HPV Story Descriptions.

*Video 1:* Provider-Patient Story at Clinic #1 – 1 min, 8 s. This video includes one Caucasian woman in her early 20′s discussing HPV vaccination with her Black female healthcare provider. The young woman admits to being sexually active but not concerned about HPV because she only has one partner. The physician discusses how she can still contract HPV. The patient then asks. “don’t people usually get the vaccine when they are kids?” and the physician admits that this is the case but that if not already vaccinated, the HPV vaccine is recommended for or all sexually active adults of the patient’s age. The physician strongly recommends vaccination, and we finish with the tag lines: “Ask your doctor about HPV,” “Tell your friends to ask their doctors,” and “Protect yourself against cervical cancer by vaccinating against HPV.”.

*Video 2:* Mother-Daughter Story – 1 min, 17 s. This video consists of one Latina daughter in her early 20′s and her Latina mother in her 40′s. The daughter enters the kitchen where her mother is sitting at the kitchen table and asks, “Why do you look so sad?” The mother replies that “Tia (Aunt) Maria” was diagnosed with cervical cancer due to HPV. After empathizing with her mother, the daughter points out that she just learning about the HPV vaccine in her college class. They move on to discuss the safety of the vaccine and the daughters desire to receive the vaccine. Towards the end, the mother reconsiders her position about non-vaccination.

*Video 3:* Rural Location Story – 1 min, 18 s. This video includes two Caucasian female friends who are high school seniors. They are unpacking their truck during the summer as they prepare to go to the river in a rural town. One girl is a current soccer coach who had a doctor’s appointment that morning for her TB shot when her doctor mentioned the HPV vaccine. The second girl asks, “What’s that?” and the initial actress responds by explaining the caners related to HPV and the number of shots you must receive as an adult to become vaccinated. The friend then discloses “My uncle had oral cancer and it was bad. Maybe I should look into getting the shot.” The girl supports her friend and says, “being protected from cancer is totally worth it.” Tag lines: “Don’t wait to vaccinate.” “Protect yourself from HPV-related cancers.” “Protect your partner too.”.

*Video 4:* Story Montage #1 – 2 min, 16 s. This video includes a montage of HPV vaccine decision stories from the point of view of two ethnically diverse fathers and teenage sons and two ethnically diverse mothers and teenage daughters. Two ethnically diverse healthcare providers offer counter arguments and health information about HPV, cancer, vaccine safety, and numbers of shots in the series. Tag line: “Ask your doctor about HPV vaccination.”.

*Video 5:* Montage #2 – 48 s. In this short video, a series of young adult men and women address the camera providing information about HPV, HPV-related cancers, and the HPV vaccination. Tag lines: “Don’t wait to vaccinate,” “Protect yourself against HPV-related cancers,” and “Protect your partner too.”.

*Video 6:* Provider-Patient Story at Clinic #2 – 1 min, 39 s. This video consists of one Latina woman in her early 20′s discussing HPV vaccination with her Caucasian female healthcare provider. The young woman explains that her mother didn’t approve of the HPV vaccine. When asked “Why?” she gets embarrassed, laughs and says, “Well, I don’t think that she likes the idea of me having sex.” The physician directs the young woman to discuss her own understanding of the vaccine and then provides information about protection against HPV-related cancers. The young woman invokes her mother’s concerns again and saying that the vaccine is “too new.” The physician again provides a fact-based rebuttal to this, discusses the safety of the vaccine, and calls upon her own role as mother saying that “in fact, I had my 11-year-old daughter vaccinated just last month. I wouldn’t give it to my daughter unless it was safe.” The scene ends with the young woman agreeing to receive the vaccine during the visit. Tag lines: “Ask your doctor about HPV,” “Tell your friends to ask their doctors,” and “Protect yourself against cervical, oral and anal cancer by vaccinating against HPV.”.
